# [Retracted] Hypoxia-induced expression of CXCR4 favors trophoblast cell migration and invasion via the activation of HIF-1α

**DOI:** 10.3892/ijmm.2025.5528

**Published:** 2025-04-07

**Authors:** Zhan Zhang, Pengyun Li, Yan Wang, Huan Yan

Int J Mol Med 42: 1508-1516, 2018; DOI: 10.3892/ijmm.2018.3701

Following the publication of the above paper, it was drawn to the Editor's attention by a concerned reader that, regarding the Transwell migration and invasion assay experiments shown in Figs. 3A, 4C, 5C and G, a number of the individual panels contained overlapping sections of data, both within and across the figure parts, such that data which were intended to show the results from differently performed experiments had apparently been derived from the same original sources.

In view of the fact that these figures were assembled erroneously, the Editor of *International Journal of Molecular Medicine* has decided that this paper should be retracted from the Journal on account of a lack of confidence in the presented data. After having been in contact with the authors, they accepted the decision to retract the paper. The Editor apologizes to the readership for any inconvenience caused.

